# Application of dexamethasone combined with tranexamic acid in perioperative period of total hip arthroplasty

**DOI:** 10.1097/MD.0000000000031223

**Published:** 2022-10-21

**Authors:** Fulin Li, Xiao Huang, Wenhui Liu, Wenwen Huang, Chaoqun Wang, Dong Yin

**Affiliations:** a Department of Joint Surgery and Sports Medicine, The People’s Hospital of Guangxi Zhuang Autonomous Region, Nanning, China.

**Keywords:** dexamethasone, THA, tranexamic acid

## Abstract

To evaluate the efficacy and safety of dexamethasone (DEXA) combined with tranexamic acid (TXA) in the perioperative period of total hip arthroplasty.

**Materials and Methods:**

A total of 100 cases were randomly divided into 2 groups (50 cases per group). All patients were given 15 mg/kg TXA before skin incision and 3 hours later. Patients in the intervention group (TXA + DEXA group) were given 20 mg dexamethasone intravenously after the onset of anesthesia, and the same dose of DEXA was administered again 24 hours later. Patients in the placebo group (TXA group) were only given the same dose of normal saline. Postoperative c-reactive protein and interleukin-6, postoperative nausea and vomiting, fatigue visual analogue scale score, postoperative length of stay, range of motion, and consumption of analgesic and antiemetics were statistically analyzed in the 2 groups.

**Results:**

The levels of c-reactive protein and interleukin-6 in the TXA + DEXA group were lower than those in the TXA group at 24, 48, 72 hours post-operatively (*P* < .001). Walking pain scores in the TXA + DEXA group were also significantly lower than those in the TXA group at 24 and 48 hours (*P* < .001); rest pain scores were lower at 24 hours (*P* < .001). Compared with the TXA group, the incidence of nausea VAS, postoperative nausea and vomiting, fatigue, analgesia and antiemetics consumption, postoperative length of stay, and range of motion were lower in the TXA + DEXA group (all *P* < .05), while there were no significant differences in postoperative hematocrit, total blood loss, and complications (*P* > .05).

**Conclusion:**

The combination of TXA (15 mg/kg; before skin incision and 3 hours later) and DEX (20 mg dexamethasone intravenously after the onset of anesthesia, and again 24 hours later) is an effective and safe strategy for patients undergoing total hip arthroplasty.

## 1. Introduction

Total hip arthroplasty (THA) is 1 of the most cost-effective treatments used for end-stage hip disease. It can significantly improve the function of the hip joint and patients’ quality of life,^[1]^ but may also cause postoperative inflammatory reaction, severe pain, postoperative nausea and vomiting (PONV), fatigue,^[2–4]^ reduce the hospitalized patients’ satisfaction,^[5]^ and hinder the quick recovery of the postoperative function.^[6]^ The management of postoperative inflammation is of great benefit to the enhanced recovery of THA.^[7,8]^

As an analog of lysine, tranexamic acid (TXA) can competitively inhibit the activation of plasminogen and the binding of plasminogen to fibrin, thereby inhibiting fibrinolysis.^[9]^ Numerous studies have suggested that TXA can significantly reduce blood loss and blood transfusion requirements, thus it become part of blood protection management during perioperative joint replacement,^[10,11]^which is the guideline for blood protection during the perioperative period of joint replacement. In addition, some studies pointed out that TXA can effectively inhibit postoperative inflammatory response and reduce postoperative pain by inhibiting the activation of plasminase.^[12,13]^ Nowadays, joint surgery is vigorously promoting the construction of accelerated rehabilitation wards, and multimodal analgesia is the core component, which also includes the suppression of postoperative inflammation is.^[14,15]^

Dexamethasone (DEXA) is a medium and long-acting glucocorticoid with strong anti-inflammatory effects. DEXA inhibits prostaglandin production by reducing the expression of cyclooxygenase 2. In this way, the expression of cell adhesion factors and the transcription of related cytokine genes can be inhibited. Neutrophils and macrophages can be inhibited to exudate to the inflammatory site and the activity of these cells can be inhibited, and their adhesion and aggregation on vascular endothelial cells in the inflammatory area can be reduced.^[16,17]^ It has been widely used in perioperative management of various surgeries, including THA. Previous studies have shown that DEXA can reduce inflammatory responses, prevent PONV, and relieve postoperative pain and fatigue in patients with THA.^[18,19]^ However, at present, the optimal dose and administration time of DEXA before THA remain unclear.^[20,21]^ In addition, only a few studies reported on the application of DEXA combined with TXA in THA.

Herein, we conducted a randomized controlled trial to compare the efficacy and safety of TXA + DEXA in THA.

## 2. Materials and Methods

### 2.1. Research design

This study was approved by the Institutional review Committee of The People’s Hospital of Guangxi Zhuang Autonomous Region and registered in the International Clinical Trial Registry (Study Registry ChiCTR2000039801). Inclusion criteria were: unilateral THA; sign the informed consent; age > 18 years old or < 75 years old. Exclusion criteria were: allergy to DEXA; age ≤ 18 years old or ≥ 75 years old; use of any glucocorticoids within 3 months or any strong opioids within 1 week before surgery; history of severe heart disease (NYHA > 2), liver and kidney failure, systemic rheumatic diseases (rheumatoid arthritis, ankylosing spondylitis, systemic lupus erythematosus); ipsilateral hip surgery history; lack of cognitive function or normal sensation; loss to follow-up.

A total of 100 patients were randomly divided into the intervention group (TXA + DEXA group) and the placebo group (TXA group), with 50 patients in each group. According to the order of patients’ admission, 100 random numbers were generated on the computer by SPSS 24.0 software from 1 to 100. The random numbers were sorted from small to large, and the rank of random numbers was 1 to 50 as the control group (TXA group) and 51 to 100 as the intervention group (TXA + DEXA group).A random allocation sequence was concealed in opaque sealed envelopes that were only opened before surgery. All patients were treated with 15 mg/kg IV-TXA before skin incision and 3 hours later. All patients in the intervention group intravenously received 20 mg DEXA after anesthesia, and the same dose was administered again 24 hours after the operation. Patients in the placebo group were intravenously given the same dose of normal saline immediately after anesthesia, and the injection was repeated 24 hours after the operation. During this study, neither the researcher nor the patient knew which group each subject was divided into, nor which group received the trial treatment.

### 2.2. Operation, anesthesia, and postoperative care

All surgeries were performed by the same group of senior joint surgeons, with an anterolateral incision approach and biotype prosthesis. All operations were evaluated by an anesthesiologist and performed under general anesthesia. In order to control the variable factors, patients were not treated with nerve block or patient-controlled intravenous analgesia during the perioperative period and were subcutaneously injected with low molecular weight heparin 6 hours after the operation. Deep vein thrombosis was detected by Doppler ultrasound 1 month after discharge. The walking and rest pain was assessed using a visual analogue scale (VAS, 0-no pain, 10-unbearable pain): if VAS levels were between 4 and 6, oxycodone was given orally at 10 mg, Q8h; if the pain was greater than 6, 100 mg of tramadol was injected intramuscularly. The VAS (VAS, 0-for nausea, 10-the most severe imaginable) was used to assess the degree of nausea. If the VAS score exceeded 6, 10 mg of metoclopramide was given as a first-line antiemetic rescue; if nausea persisted within 30 minutes, 5 mg of ondasetron was used as second-line antiemesis aid.

The total blood loss was calculated according to the general formula, and the blood transfusion indications were strictly controlled only when the hemoglobin level was < 70 g/L or 70 to 00 g/L, accompanied by anemia symptoms such as mental state, dizziness, etc.

### 2.3. C-reactive protein (CRP), interleukin-6 (IL-6), VAS pain score, nausea VAS score, and POVN incidence

CRP, IL-6, VAS pain score, nausea VAS score, and POVN incidence were recorded at 24, 48, and 72 hours postoperatively. The primary outcome variables of this study were VAS, CRP and IL-6 after operation, and other outcome variables were secondary observation indexes. The total number and dose of postoperative analgesics (oxycodone, tramadol hydrochloride) and antiemetic salvage drugs (metoclopramide, Ondaasetron) were recorded. Fatigue was assessed preoperatively and at discharge using the 10-point numerical scoring scale (NRS, 1-FIT, 10-fatigue).^[22]^ Activity (ROM) was assessed by the nurse with a goniometer before surgery and at discharge, and postoperative length of stay (p-LOS) and complications were carefully recorded.

### 2.4. Statistical analysis

Sample size calculations were analyzed by PASS 2011 (NCSS, LLC. Kaysville, UT, available at https://www.ncss.com/software/pass/) software based on a 2-sample *t* test. All data were analyzed by SPSS 22.0 (SPSS 22.0 Inc, USA, available at https://www.ibm.com/analytics/spss-statistics-software). *T* test or Wilcoxon Mann-Whiney *U* test was used to analyze quantitative data, and Pearson Chi-square test or Fisher exact test was used to analyze qualitative comparative data. *P* < .05 was considered to be statistically significant. With the power of 0.90 and the significant level of 0.05, 43 patients per arm were required in the study. Considering the dropping out, 10% to 15% of the sample size should be increased at least. In this study, we calculated that at 15%. Therefore, the sample size of 50 cases each group was required for this trial, so the total sample size was 100 cases.

## 3. Results

### 3.1. Baseline characteristics

**Figure 1.**
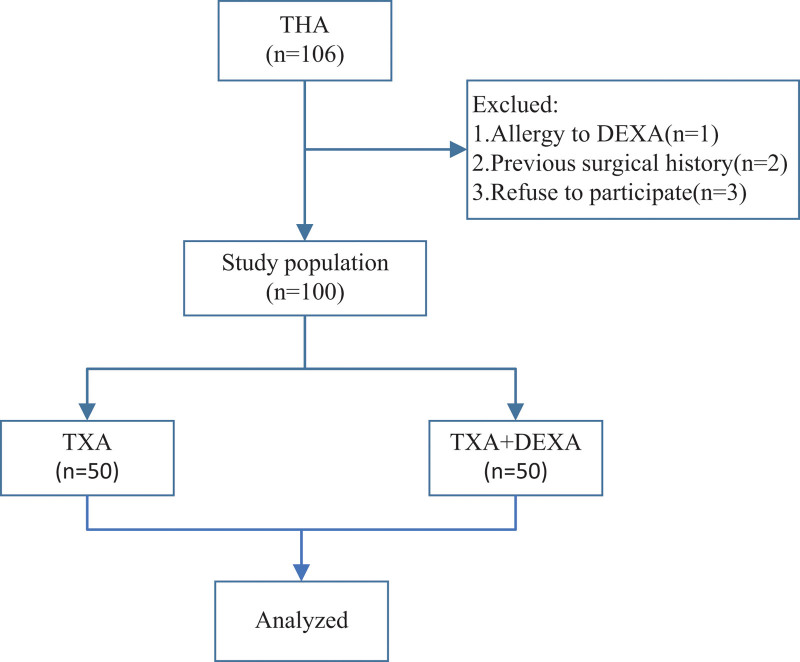
Schematic diagram of the patient study process.

During recruitment, 106 patients underwent primary unilateral THA. Six patients were not eligible for inclusion, including 1 patient who was allergic to DEXA, 2 patients with a history of surgery, and 3 patients who refused to participate. Finally, 100 patients were included in the study, and all patients completed the follow-up (Fig. 1). There was no difference in baseline characteristics between the 2 groups (Table 1).

**Table 1:** Demographic data of the patients receiving THA.

Variables	TXA	DEXA + TXA	*P*
N	50	50	–
Age (yrs)	64.02 ± 5.82	64.42 ± 5.23	0.72
Gender (M/F)	21/29	22/28	0.84
Height (cm)	1.62 ± 0.08	1.63 ± 0.09	0.40
Weight (kg)	64.39 ± 8.30	65.36 ± 8.73	0.53
BMI (kg/m^2^)	24.63 ± 2.77	24.64 ± 3.02	0.99
Hypertension (Y/N)	11/39	13/37	0.64
Diabetes (Y/N)	2/48	3/47	0.65
Etiology (ONFH/OA/DDH)	27/14/9	24/16/10	0.61
Preoperative CRP	7.86 ± 2.67	7.54 ± 2.18	0.54
Preoperative IL-6	2.33 ± 1.38	2.68 ± 1.60	0.23
Preoperative rest VAS	5.44 ± 0.86	5.36 ± 1.12	0.69
Preoperative motive VAS	7.98 ± 0.87	8.04 ± 0.88	0.73
Preoperative ICFS score	61.76 ± 5.86	63.26 ± 4.64	0.16
Preoperative HCT	39.64 ± 1.63	39.88 ± 1.47	0.41
Preoperative Hb	126.12 ± 8.90	125.28 ± 7.53	0.61
Preoperative ROM	90.48 ± 4.10	91.14 ± 3.51	0.39

Table shows demographic data of the patients receiving THA.

BMI = body mass index, CRP = c-reactive protein, DDH = development displasia hip, Hb = hemoglobin, HCT = hematocrit, ICFS = identity-consequence-fatigue-scale, IL-6 = interleukin-6, OA = osteoarthritis, ONFH = osteonecrosis of the femoral head, ROM = range of motion., VAS = visual analogue scale.

### 3.2. CRP and IL–6

**Figure 2.**
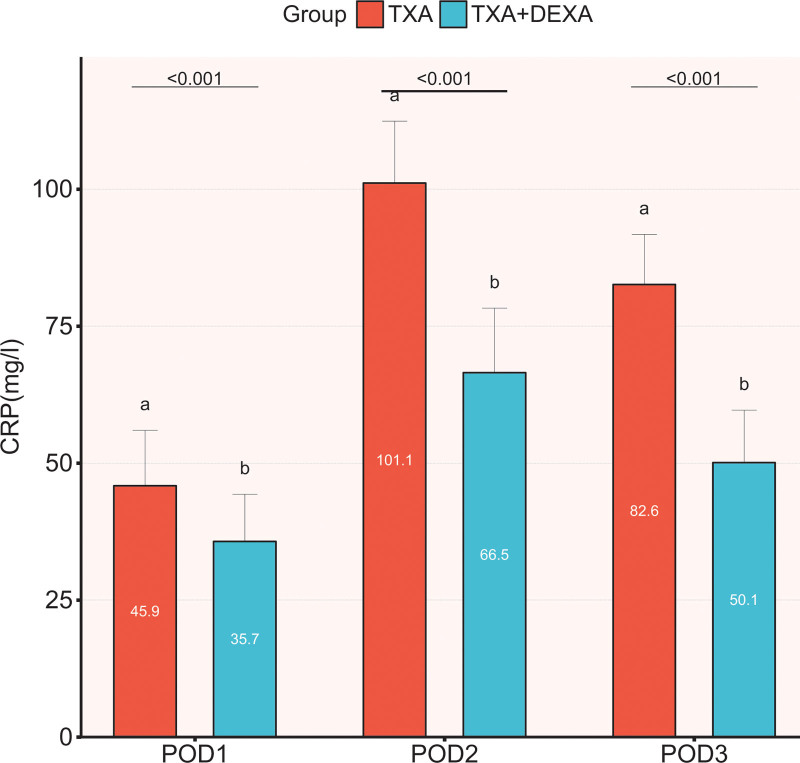
The comparison of CRP between the 2 groups on POD 1, 2, and 3. The Student’s *t* test was performed to detect the difference between the groups.* *P* < .05. Figure created using Graphpad prism (Graphpad prism: Mapping Software and Statistical tools. Version 8.2.1; 2019; available at https://www.graphpad.com/). CRP = c-reactive protein.

**Figure 3.**
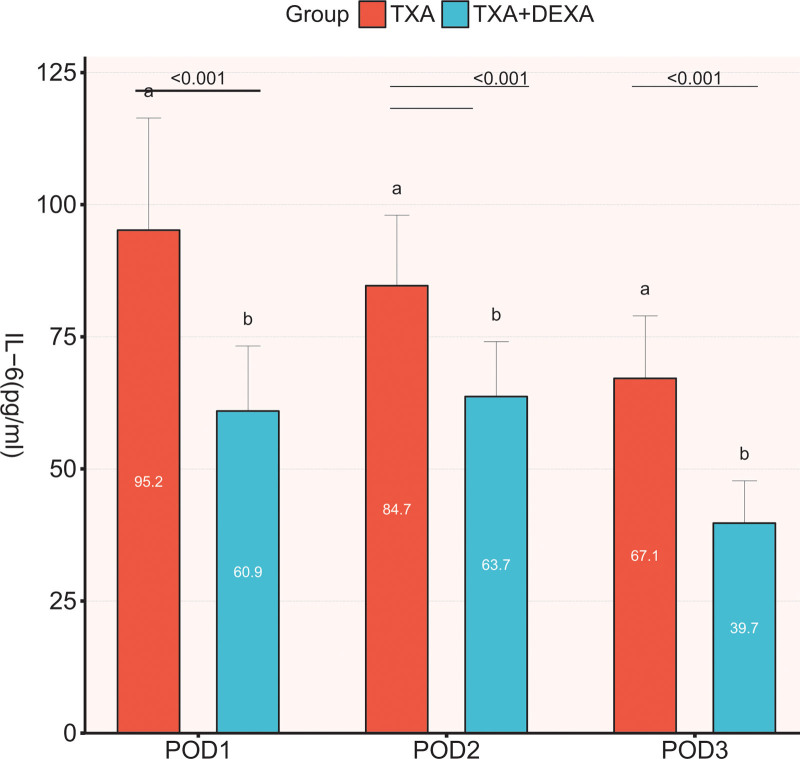
The comparison of IL-6 between the 2 groups on POD 1, 2, and 3. The Student’s *t* test was performed to detect the difference between the groups.* *P* < .05. Figure created using Graphpad prism (Graphpad prism: Mapping Software and Statistical tools. Version 8.2.1; 2019; available at https://www.graphpad.com/). IL-6 = interleukin-6.

CRP and IL-6, as the markers of acute inflammation, were elevated in all patients after surgery. The mean CRP level of patients in both groups reached the peak at 48 hours after surgery; yet, the mean CRP level in the TXA + DEXA group was significantly lower at 24, 48, and 72 hours after surgery (*P* < .001) than that in the TXA group (Fig. 2). The average level of IL-6 in the TXA group reached the peak 24 hours after the operation, while in the TXA + DEXA group, it peaked at 48 hours after surgery. Similarly, the average level of IL-6 was significantly lower in TXA + DEXA group compared to the TXA group (*P* < .001) at 24 hours, 48 hours (*P* < .001), and 72 hours (*P* < .001) after surgery (Fig. 3).

### 3.3. Pain-related information

#### 3.3.1. VAS score.

**Figure 4.**
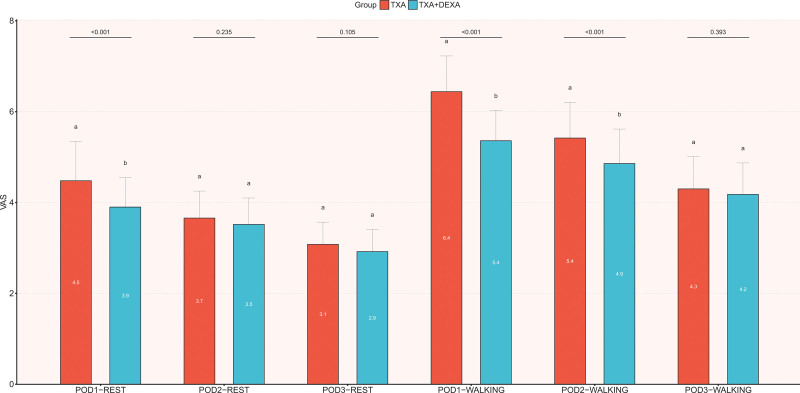
The comparison of VAS of pain at rest and walking between the 2 groups on POD 1, 2, and 3. The Student’s *t* test was performed to detect the difference between the groups.* *P* < .05. Figure created using Graphpad prism (Graphpad prism: Mapping Software and Statistical tools. Version 8.2.1; 2019; available at https://www.graphpad.com/). VAS = visual analogue scale.

Compared with the preoperative, the postoperative pain during rest and walking was significantly reduced in all patients. Postoperative pain during rest at 24 hours (*P* < .001), and postoperative pain during walking at 24 hours (*P* < .001) and 48 hours (*P* < .001) were significantly lower in the DEXA + TXA group than in the TXA group. However, VAS scores for rest at 48 hours after surgery and VAS scores for rest and walking at 72 hours after surgery revealed no significant difference between the 2 groups (*P* > .05) (Fig. 4).

#### 3.3.2. Analgesic medications.

Thirty patients in the TXA + DEXA group and less than 37 patients in the TXA group needed oxycodone; however, the difference was not statistically significant (*P* = .14). The cumulative consumption of oxycodone was 530 mg in the TXA + DEXA group and 740 mg in the TXA group, with no significant difference (*P* = .08). For tramadol hydrochloride, the number of patients required in the TXA + DEXA group was significantly different compared with the TXA group (*P* < .001), and the cumulative consumption of the TXA + DEXA group was also lower (*P* = .004)(**Table 2**).

**Table 2:** The requirement of rescue treatment between the 2 groups.

Variables	TXA	DEXA + TXA	*P*
Oxycodone			
N	37/50	30/50	.14
Total dose (mg)	740	530	.08
Tramadol			
N	19/50	9/50	.00
Total dose (mg)	3600	1100	.004
Metoclopramide			
N	13/50	3/50	.006
Total dose (mg)	180	40	.009
Ondansetron			
N	4/50	1/50	.17
Total dose (mg)	20	5	.17

Table shows the total amount and number of patients who consumed oxycodone, tramadol, metoclopramide, and ondasetron.

DEXA = dexamethasone, TXA = tranexamic acid.

### 3.4. PONV and antiemetic drugs

In the TXA + DEXA group, 3 patients needed metopramide compared with the thirteen patients in the TXA group (*P* = .006). The cumulative consumption of metoplatin in the TXA + DEXA group was 40 mg, and that in the TXA group was 180 mg; the observed difference was significantly different (*P* = .009). Moreover, in the TXA + DEXA group, 1 patient required ondansetron compared to 4 patients in the TXA group, but the difference was not statistically significant (*P* = .17). Meanwhile, there was no significant difference in the cumulative consumption of ondansetron between the 2 groups (*P* = .17) (Table 2).

VAS scores of postoperative nausea were significantly lower in the TXA + DEXA group than in the TXA group, and the difference was statistically significant (*P* = .025). In terms of PONV incidence, DEXA + TXA was 6% (3/50) and 24% (12/50) in the TXA group; the observed difference was statistically significant (*P* = .011) (Table 3).

**Table 3:** The clinical effect and complications.

Variables	TXA	DEXA + TXA	*P*
PONV	13/50	3/50	.006
VAS-nausea	1.90 ± 1.80	1.20 ± 1.23	.025
Post-ICFS	78.86 ± 10.18	68.66 ± 8.68	.00
ROM	93.80 ± 3.88	95.98 ± 2.82	.002
Blood transfusion rate	5/50	4/50	.73
Total blood loss	934.80 ± 103.50	942.20 ± 111.27	.73
Post-LOS (d)	5.56 ± 0.81	5.02 ± 0.52	.00
Wound problems	2/50	1/50	.56
DVT/PE	0/50	0/50	–
Infection	0/50	0/50	–
Gastrointestinal Hemorrhage	0/50	0/50	–

Table shows the patient’s postoperative outcomes and complications.

DVT = Deep vein thrombosis, ICFS = Identity-Consequence-Fatigue-Scale, PE = pulmonary embolism, PONV = postoperative nausea and vomiting, Post-LOS = postoperative length of stay, ROM = range of motion, VAS = visual analogue scale.

### 3.5. Fatigue, ROM, p-LOS, and blood loss

The degree of fatigue in the TXA + DEXA group was significantly lower than that in the TXA group, and the difference was statistically significant (*P* < .001). The ROM of patients in the TXA + DEXA group was greater than that of the TXA group at discharge, and the comparison between the 2 groups was statistically significant (*P* = .002). The mean p-LOS of the TXA + DEXA and TXA groups was (5.02 ± 0.52) days and (5.56 ± 0.81) respectively (all *P* < .001). There was no statistical difference between the 2 groups in terms of total blood loss and transfusion rate (*P*1 = 0.73, *P*2 = 0.73) (Table 3).

### 3.6. Complications

No deep vein thrombosis or pulmonary embolism was found in any of patients within 1 month after discharge, according to the ultrasound. In the TXA + DEXA group, 1 patient had an incision line reaction, and 2 patients in the TXA group had incision fat liquefaction. None of the patients had a gastrointestinal hemorrhage or incision infection (Table 3).

## 4. Discussion

TXA is a synthetic lysine analogue that inhibits fibrinolysis by competitively blocking the binding site of plasmin pyrrolysine, often used in joint replacement to reduce perioperative blood loss.^[9]^ Studies have shown that fibrinolytic enzyme has an important role in connecting fibrinolytic and inflammatory systems. It can stimulate the release of cytokines of different cell types and other inflammatory mediators to promote inflammation. Therefore, TXA, as an anti-fibrinolytic drug, can indirectly exert an anti-inflammatory role.^[12,13,23]^

DEXA is a medium long-acting glucocorticoid with strong anti-inflammatory properties, widely used to reduce perioperative inflammatory response, prevent PONV, and relieve perioperative and postoperative pain and fatigue.^[24]^ A number of randomized controlled trials (RCTS) have demonstrated the efficacy of DEXA in preventing inflammatory stress without causing a wound and gastrointestinal bleeding complications during THA.^[20,21]^ However, so far, no optimal dose and optimal time of DEXA injection before THA has been proposed. Moreover, only a few studies reported on the application of DEXA combined with TXA in THA. An et al^[25]^ evaluated the efficacy and safety of combined use of TXA and DEX for anti-inflammatory and clinical outcomes after THA. Patients who underwent THA were given 20 mg IV-DEXA only when anesthesia was completed in the intervention group, and the same dose of normal saline was applied in the placebo group. All patients were given 15 mg/kg TXA before skin incision and 3 hours later, which confirmed that DEXA + TXA could relieve postoperative pain, PONV, and fatigue and did not increase the risk of postoperative complications. In our study, patients received 15 mg/kg TXA before skin incision and 3 hours later. Patients in the intervention group were given 20 mg IV-DEXA when the anesthesia was completed and 24 hours later, which was different from An et al The control group was given the same dose of normal saline. We discovered that TXA + DEXA could significantly reduce postoperative CRP and the level of IL-6, reduce PONV, postoperative pain, and fatigue, increase the effect of analgesia and anti-nausea without affecting the hemostatic effect of TXA or increasing the risk of complications such as wound infection and gastrointestinal bleeding.

As markers of acute inflammation, CRP and IL-6 levels show similar dynamic changes during the inflammatory response. Previous studies have confirmed that local and systemic inflammatory responses are closely related to early postoperative rehabilitation and complications.^[27,28]^ In our study, 20 mg DEXA was intravenously administered before surgery and 24 hours later, and the postoperative CRP and IL-6 levels were significantly reduced, which was consistent with previous studies.^[8,17,20,21]^ Nevertheless, the optimal dose, duration of treatment, and route of administration of DEXA remain controversial.^[29]^ In our study, DEXA combined with TXA further significantly reduced the levels of CRP and IL-6 (at 24, 48, and 72 hours after surgery), alleviated postoperative pain, and accelerated rapid recovery. In our study, unlike most studies using TXA or DEXA alone, we hypothesized that DEXA combined with TXA had synergistic and additional benefits in reducing postoperative inflammatory responses compared with TXA alone. Moreover, our data confirmed the hypothesis that combined strategies could reduce CRP and IL-6 levels.

Previous studies have confirmed the anti-inflammatory and analgesic effects of TXA.^[12,13]^ It is worthy to note that some patients still had pain symptoms after surgery. Therefore, we considered whether DEXA combined with TXA could potentially compensate for the lack of anti-inflammatory effects of TXA, further reducing pain and accelerating the recovery of patients. The analgesic effect of DEXA is to block the pathway of cyclooxygenase and lipase in the inflammatory chain reaction by inhibiting the phospholipase. In addition, DEXA can also inhibit the level of 35-bradykinin in tissue, release neuropeptides from the nerve endings, and reduce the inflammation and pain in the tissue.^[16]^ Previous studies have confirmed the good analgesic effect of DEXA in the perioperative period of THA. Lei et al^[17]^ examined 210 patients who underwent THA and were randomly divided into 3 groups: Group A was the blank group; group B was given 10 mg IV-DEXA after anesthesia and then again after 3 hours; Group C was given 10 mg IV-DEXA after anesthesia and then again after 3 and 24 hours. Our results showed that the additional DEXA could further reduce the pain and reduce the application of analgesic drugs. Based on the previous study of analgesic drug consumption, we considered that a single application of 20 mg IV-DEXA might not be enough for analgesic effect, so we injected a second dose again after 24 hours. We hypothesized that increasing the dose of DEXA might provide additional analgesic effects. In our study, pain scores were lower at 24 and 48 hours of walking and 24 hours of rest after surgery. In addition, the consumption of oxycodone and tramadol hydrochloride was lower, which further confirmed that the short-term efficacy of increasing DEXA dose could further reduce pain and also compensate for the deficiency of TXA as an anti-inflammatory. It is important to note that the long-term effects of DEXA on relieving pain have not yet been proved.

The antiemetic effect of DEXA has been confirmed, mainly by regulating prostaglandin synthesis or inhibiting endogenous opioid release.^[30,31]^ In the current study, using a single dose of a glucocorticoid to reduce the incidence of PONV was supported and recommended.^[20,21]^ We also hypothesized that patients would receive PONV remission 24 hours after surgery if the additional dose was supplied compared with single-dose treatment. In our study, the incidence of nausea VAS score and PONV in the TXA + DEXA group was significantly lower than that in the TXA group (*P* < .05), and the number of metoclopramide and total cumulative consumption were also significantly lower than that in the TXA group (*P* < .05). This data suggests that 1 additional dose of DEXA 24 hours after surgery can effectively reduce the incidence of PONV.

Postoperative fatigue is a common clinical symptom, which often leads to delay in early recovery and affects patients’ initiative rehabilitation.^[20,21,32]^ In their meta-analysis of the relationship among pain, sleep, and fatigue, Whibley et al^[32]^ pointed out that these 3 factors influence each other and interact. Only when each factor is positively controlled, this virtuous circle can be established to promote early recovery of patients, shorten the average length of stay in the hospital, and improve patients’ satisfaction. In our study, Identity-consequence-fatigue-scale was used to assess fatigue, and the average p-LOS was calculated. The results showed that postoperative Identity-consequence-fatigue-scale scores and p-LOS in the TXA + DEXA group were significantly reduced compared with the TXA group (*P*1 < 0.001, *P*2 < 0.001).

The optimal approach and dose of DEXA before THA are still controversial. Some studies have identified a dose range of 10 to 40 mg for primary THA.^[26]^ Yet, patients given low doses of DEXA may still suffer from pain, fatigue, and PONV. Therefore, this study was based on the assumption that the effect of low-dose DEXA still did not meet the anti-inflammatory requirements, and its half-life was fully considered to be about 24 hours. Accordingly, it is reasonable to administer 20 mg DEXA again 24 hours after surgery. As for the dose of IV-TXA, the guidelines recommend that 15 to 20 mg/kg can be given, depending on the patients. In our previous studies, the dose of 15 mg/kg of TXA achieved satisfactory clinical effects. Our study showed that DEXA combined with TXA could effectively reduce postoperative inflammation and pain, without affecting the hemostatic effect of TXA.

Although DEXA is widely used in the perioperative period of THA, it is still controversial whether DEXA might increase the risk of adverse reactions.^[13,17,29]^ DEXA has been applied again before and 24 hours after surgery in our study. One patient had a thrum reaction in the TXA + DEXA group, and in the TXA group, 1 patient had incision fat liquefaction, and the other had a thrum reaction. During observation, no incision infection or gastrointestinal bleeding and no thrombosis was found in any of patients. However, it should be pointed out that large-scale prospective studies are still needed to assess the safety of DEXA in perioperative application of THA and clinical application.

This study has some limitations. First, the follow-up time was too short. Three-month follow-up was insufficient to fully evaluate the efficacy and safety of TXA + DEXA. Second, this study has a relatively small sample size. Third, the optimal combination dose and timing of TXA + DEXA remain unclear and need to be further studied.

## 5. Conclusion

Our data suggest that DEXA combined with TXA can significantly reduce postoperative CRP and IL-6 levels, reduce postoperative pain, improve the incidence of POVN, increase the analgesic and antiemetic effects, reduce postoperative fatigue, shorten the average p-LOS, and improve ROM without affecting the hemostatic effect of TXA or increasing the risk of perioperative complications of THA.

## Authors’ contributions

Fulin-Li performed the data collection and analysis and participated in manuscript writing. Xiao Huang, Wenhui-Liu, Wenwen-Huang and Chaoqun-Wang performed the database setup and statistical analysis. Dong Yin performed the operations, participated in the study design and coordination, and helped to draft the manuscript. All of the authors have read and approved the final manuscript.

**Data curation:** Fulin-Li, Xiao Huang, Wenwen Huang, Chaoqun-Wang, Dong Yin.

**Formal analysis:** Xiao Huang, Chaoqun-Wang, Dong Yin.

**Funding acquisition:** Fulin-Li.

**Investigation:** Fulin-Li, Wenwen Huang.

**Methodology:** Fulin-Li, Wenwen Huang, Dong Yin.

**Resources:** Wenhui Liu.

**Software:** Fulin-Li, Wenhui Liu.

**Supervision:** Wenhui Liu.

**Writing – original draft:** Fulin-Li, Dong Yin.

**Writing – review & editing:** Fulin-Li, Dong Yin.
